# KMT2C methyltransferase domain regulated INK4A expression suppresses prostate cancer metastasis

**DOI:** 10.1186/s12943-022-01542-8

**Published:** 2022-03-30

**Authors:** Tanja Limberger, Michaela Schlederer, Karolina Trachtová, Ines Garces de los Fayos Alonso, Jiaye Yang, Sandra Högler, Christina Sternberg, Vojtech Bystry, Jan Oppelt, Boris Tichý, Margit Schmeidl, Petra Kodajova, Anton Jäger, Heidi A. Neubauer, Monika Oberhuber, Belinda S. Schmalzbauer, Sarka Pospisilova, Helmut Dolznig, Wolfgang Wadsak, Zoran Culig, Suzanne D. Turner, Gerda Egger, Sabine Lagger, Lukas Kenner

**Affiliations:** 1 Division of Experimental and Translational Pathology, Department of Pathology, Medical University of Vienna, 1090 Vienna, Austria; 2 CBmed-Center for Biomarker Research in Medicine GmbH, 8010 Graz, Austria; 3 Central European Institute of Technology, Masaryk University, Brno, 62500 Czech Republic; 4 Christian Doppler Laboratory for Applied Metabolomics, 1090 Vienna, Austria; 5 Division of Nuclear Medicine, Department of Biomedical Imaging and Image-Guided Therapy, Medical University of Vienna, 1090 Vienna, Austria; 6 Unit of Laboratory Animal Pathology, University of Veterinary Medicine Vienna, 1210 Vienna, Austria; 7 Institute of Biochemistry, Christian-Albrechts-University Kiel, 24118 Kiel, Germany; 8 Institute of Animal Breeding and Genetics, University of Veterinary Medicine Vienna, 1210 Vienna, Austria; 9 Institute of Pharmacology and Toxicology, University of Veterinary Medicine Vienna, 1210 Vienna, Austria; 10 Institute of Medical Genetics, Medical University of Vienna, 1090 Vienna, Austria; 11 Department of Urology, Innsbruck Medical University, 6020 Innsbruck, Austria; 12 Department of Pathology, University Cambridge, Cambridge, UK; 13 CEITEC, Masaryk University, Brno, Czech Republic; 14 Ludwig Boltzmann Institute Applied Diagnostics, 1090 Vienna, Austria

**Keywords:** Prostate cancer, Senescence, Metastasis, KMT2C, MYC, p16^INK4A^

## Abstract

**Background:**

Frequent truncation mutations of the histone lysine N-methyltransferase *KMT2C* have been detected by whole exome sequencing studies in various cancers, including malignancies of the prostate. However, the biological consequences of these alterations in prostate cancer have not yet been elucidated.

**Methods:**

To investigate the functional effects of these mutations, we deleted the C-terminal catalytic core motif of *Kmt2c* specifically in mouse prostate epithelium. We analysed the effect of *Kmt2c* SET domain deletion in a *Pten*-deficient PCa mouse model in vivo and of truncation mutations of *KMT2C* in a large number of prostate cancer patients.

**Results:**

We show here for the first time that impaired KMT2C methyltransferase activity drives proliferation and PIN formation and, when combined with loss of the tumour suppressor PTEN*,* triggers loss of senescence, metastatic dissemination and dramatically reduces life expectancy. In *Kmt2c*-mutated tumours we show enrichment of proliferative MYC gene signatures and loss of expression of the cell cycle repressor p16^INK4A^. In addition, we observe a striking reduction in disease-free survival of patients with *KMT2C*-mutated prostate cancer.

**Conclusions:**

We identified truncating events of *KMT2C* as drivers of proliferation and PIN formation. Loss of PTEN and KMT2C in prostate cancer results in loss of senescence, metastatic dissemination and reduced life expectancy. Our data demonstrate the prognostic significance of *KMT2C* mutation status in prostate cancer patients. Inhibition of the MYC signalling axis may be a viable treatment option for patients with KMT2C truncations and therefore poor prognosis.

**Supplementary Information:**

The online version contains supplementary material available at 10.1186/s12943-022-01542-8.

## Introduction

Prostate cancer (PCa) ranks as the second most frequently diagnosed malignancy in men worldwide and is expected to surpass even lung cancer incidence levels within the next decade [1, 2]. Diagnosis and therapy are challenged by enormous inter-tumour heterogeneity regarding clinical, morphological, and molecular features [3]. While patients with localized or regional disease have an excellent prognosis, metastatic PCa remains largely incurable [4]. Therefore, therapeutic strategies must be tailored to the individual risk of the patient to avoid overtreatment of low-risk tumours while ensuring rapid and decisive intervention in high-risk cases. To better stratify PCa and to advance the development of new therapies, a deeper understanding of the genetic and epigenetic events responsible for the progression and metastatic spread of PCa is urgently needed.

Multiple key pathways of prostate tumorigenesis have already been identified. Inactivating mutations of the phosphatase and tensin homolog (*PTEN*) tumour suppressor gene rank among the most common alterations observed in PCa [5]. Loss of PTEN results in the aberrant activation of the phosphoinositide 3-kinase (PI3K) – AKT signalling pathway, which uncouples proliferation, survival and metabolism from external growth-stimulatory signals [6]. However, loss of PTEN has also been shown to induce cellular senescence. This form of cell cycle arrest, which can be triggered upon oncogenic stress, is usually mediated via the p16^INK4A^-RB and the p14^ARF^-p53-p21^CIP1^ pathways and has previously been shown to act as a barrier to metastatic transformation in PCa [7]. Besides inactivation of tumour suppressor genes, the amplification and overexpression of oncogenes is similarly known to play a crucial role in prostate tumorigenesis. The frequently altered androgen receptor (AR) signalling axis is the most well-studied pathway in the context of PCa. However, other key effectors, such as the activation of the proto-oncogene MYC, have also been found to be fundamental to PCa progression [8].

Besides genetic mutations, several epigenetic alterations, including DNA and histone modifications, have been identified in clinical PCa samples. Only recently has the pivotal importance of epigenetic reprogramming as a driver of carcinogenesis been widely recognized [9, 10]. Large cancer genome sequencing projects have revealed a substantial number of alterations in epigenetic modulators [11]. The histone lysine N-methyltransferase *KMT2C*, an enzymatically active scaffold protein within the COMPASS (Complex Proteins Associated with Set1) multi-subunit complex, is the most frequently mutated gene within this group, predominantly presenting with frameshift and nonsense mutations [12, 13]. Previous studies have shown evidence of tumour suppressive roles for KMT2C and its close paralogue KMT2D, and have proposed their involvement in cellular growth, stemness and epithelial differentiation [13, 14]. However, despite the tremendous prevalence of mutations in these genes, the molecular mechanisms contributing to carcinogenic processes are still poorly understood [13]. In PCa, alterations of epigenetic modifiers and chromatin-remodelling genes occur in about 20% of tumours with a strong overrepresentation of *KMT2C* mutations (7%), suggestive of a crucial role in carcinogenesis [15]. A substantial number of mutations detected in *KMT2C* lead to loss of function of the encoded protein, whereby the catalytic domain is lost.

In this study, we investigated the effects of loss of the catalytic domain of KMT2C on the development and progression of PCa. We established a transgenic mouse model with prostate-specific deletion of either the catalytic core motif of KMT2C alone or in combination with loss of the tumour suppressor PTEN. In these models we observed that mutant KMT2C drives proliferation in vivo and triggers PCa metastasis when co-deleted with PTEN. Our data show that loss of the KMT2C catalytic core motif, mimicking the scenario in patients, results in an enrichment of the proliferative MYC gene signature and impairs p16^INK4A^-mediated cell cycle arrest in both our model system and human prostate cancers. Importantly, we show that mutated KMT2C significantly correlates with reduced disease-free survival (DFS) for PCa patients. Taken together, we identify the SET domain deletion of KMT2C as a novel driver of prostate carcinogenesis in murine models and suggest that the presence of mutated forms is a biomarker for poor outcome in PCa patients. Furthermore, our data are indicative of a possible therapeutic application through blockade of the MYC pathway.

## Results

### KMT2C SET Domain Deletion Initiates Formation of Prostatic Intraepithelial Neoplasia In Vivo

**Fig. 1:**
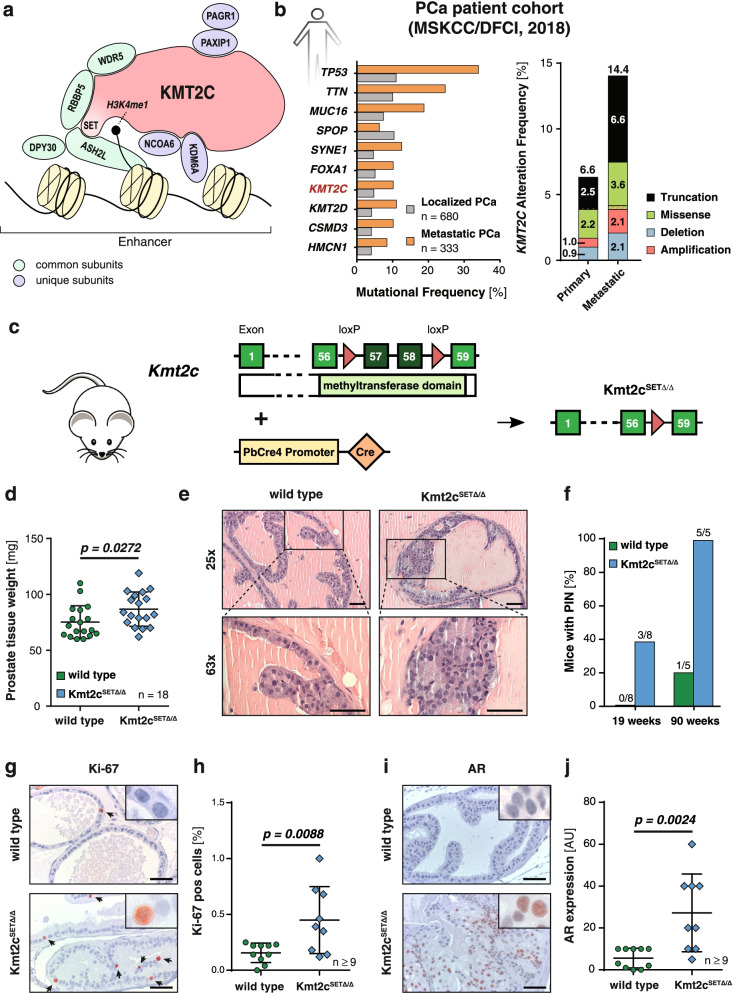
Mutant KMT2C induces prostate intraepithelial neoplasia. **a** Schematic representation of the multi-subunit COMPASS (Complex Proteins Associated with Set1) complex containing KMT2C. KMT2C/D acts as a scaffold to bind multiple subunits unique to KMT2C or KMT2D containing COMPASS-like complexes (PAX-interacting protein 1 (PAXIP1), PAXIP1-associated glutamate-rich protein 1 (PAGR1), nuclear receptor coactivator 6 (NCOA6), lysine specific demethylase 6A (KDM6A)) as well as proteins common to all KMT2 complexes (WD repeat-containing protein 5 (WDR5), Retinoblastoma-binding protein 5 (RBBP5), Set1/Ash2 histone methyltransferase complex subunit ASH2 (ASH2L), Protein dpy-30 homolog (DPY30)). The SET domain located at the C-terminal end of the protein confers the methyltransferase activity needed to methylate lysine 3 on histone 4 (H3K4me1) at enhancer regions. **b** Spectrum of *KMT2C* mutations in the MSKCC/DFCI patient cohort comprised of 680 primary and 333 metastatic PCa samples. Data were retrieved from cBioPortal. Left panel: Top 10 most frequently mutated genes in this cohort. Right panel: Proportion of *KMT2C* alterations found in primary and metastatic PCa samples. Values within and above bars indicate alteration frequency of the respective mutation type and total percentage of altered samples, respectively. Samples with simultaneous mutations of more than one class are depicted with alternating stripes of both respective colours. **c** Scheme of the construct allowing for the conditional deletion of exon 57 and 58 within the SET domain of *Kmt2c*, which confers the methyltransferase activity, and the mutant transcript expressed specifically in prostate epithelial cells after *Cre-*mediated recombination controlled by the androgen-dependent *probasin* promoter (hereafter Kmt2c^SET∆/∆^). **d** Weight of wild type and Kmt2c^SET∆/∆^ prostates at 19 weeks p.p. (*n* = 18) **e** Representative pictures of PIN formation in a 19-week-old Kmt2c^SET∆/∆^ prostate. Wild type prostate is shown as a control. Scale bars: 50 µm. **f** Percentage of wild type and Kmt2c^SET∆/∆^ mice presenting with PIN at 19- and 90 weeks p.p. Number above the bar indicates PIN-positive/total number of analysed mice. (*n* ≥ 5) **g-h** Representative pictures of Ki-67 IHC analysis of wild type and Kmt2c^SET∆/∆^ mouse prostate tissue at 19 weeks p.p. (*n* ≥ 9) (**g**) and associated quantification (**h**). Scale bars: 50 µm. Cells positive for Ki-67 were quantified using QuPath software. **i-j** Representative pictures of AR IHC analysis of wild type and Kmt2c^SET∆/∆^ mouse prostate tissue at 19 weeks p.p. (*n* ≥ 9) (**i**) and associated quantification (**j**). Scale bars: 50 µm. Stains were semi-quantitatively analysed by a board-certified genitourinary pathologist. Arbitrary unit (AU) is a multiplication of percentage of positive cells and staining intensity (0, 1, 2, 3). **(d**, **h**, and **j)** Individual biological replicates are shown. Data are plotted as the mean ± standard deviation, and *P* values were determined by unpaired two-tailed Student’s t-tests

Whole exome sequencing studies of various human cancers have identified frequent somatic mutations in the gene encoding the histone-methyltransferase KMT2C [14]. Like other KMT2 proteins, KMT2C acts as a scaffold for the multi-subunit COMPASS complex where it regulates enhancer elements mainly through mono-methylation of lysine 3 on histone 4 (H3K4me1) via the enzymatically active SET domain located at the C-terminal end of the protein [13, 16] (Fig. 1a). To gain insight into the mutational spectrum of human PCa we analysed a cohort of 1013 patients with either localized or metastatic disease (MSKCC/DFCI cohort) [15] and found *KMT2C* to be the 7^th^ most frequently mutated gene (Fig. 1b, left panel). In contrast to previously published data of the mutational pattern of *KMT2C* in different human cancers [17], *KMT2C* mutations in the analysed PCa dataset were distributed along the gene with no apparent mutational hotspot (Supplementary Fig. 1a) in keeping with the types of mutations commonly observed in tumour suppressor genes. While the functional significance of individual missense mutations is difficult to discern, truncation mutations (nonsense, frameshift, and splice mutations), which account for the majority of the changes we detected in *KMT2C* in the MSKCC/DFCI cohort, are primarily predicted to negatively impact the C-terminal SET domain, and are twice as common in metastatic samples in this dataset (Fig. 1b, right panel). Thus, we hypothesized that mutations leading to loss of the methyltransferase activity of KMT2C play a functional role in the initiation and/or progression of PCa. To investigate the impact of impaired KMT2C methyltransferase activity in vivo we established a mouse model with prostate-specific deletion of the *Kmt2c* SET domain-encoding exons (Kmt2c^SETΔ/Δ^). Specifically, we crossed mice carrying loxP sites flanking exons 57 and 58, which encode the catalytic core motif of KMT2C [18], to mice carrying the *Cre* recombinase transgene under the control of the androgen-regulated prostate-specific probasin promoter (PbCre4) [19] (Fig. 1c). Deletion of the *Kmt2c* SET domain-encoding exons was confirmed after the onset of puberty at both genetic and transcriptional levels by Polymerase Chain Reaction (PCR) and quantitative reverse transcription—PCR (RT-qPCR), respectively (Supplementary Fig. 1b-c). RT-qPCR analysis of total *Kmt2c* mRNA expression, using primers detecting a region outside of the deleted locus, revealed comparable levels of the mutated mRNA transcripts in *Kmt2c*^SETΔ/Δ^ mouse prostates to wild type *Kmt2c* expression in control animals (Supplementary Fig. 1d). Interestingly, we found no compensatory up-regulation of the closely related paralogue *Kmt2d* (Supplementary Fig. 1e). Upon macroscopic evaluation, we discovered that the total prostate weight was significantly increased in mutant animals at 19 and 90 weeks *postpartum* (p.p.) compared to wild type controls (Fig. 1d, Supplementary Fig. 1f). Analysis of haematoxylin and eosin (H&E) stained sections of mouse prostates revealed focal areas of prostatic intraepithelial neoplasia (PIN) in Kmt2c^SET∆/∆^ mice sacrificed as early as 19 weeks p.p. increasing to full penetrance by 90 weeks p.p. (Fig. 1e-f). Immunohistochemistry (IHC) showed a significant increase in the percentage of Ki-67 positive proliferating cells in Kmt2c^SET∆/∆^ prostates (Fig. 1g-h, Supplementary Fig. 1g) and clusters of cells highly positive for AR expression, the primary driver of prostate cancer initiation and progression (Fig. 1i-j). These data suggest that loss of KMT2C catalytic activity drives proliferation and initiates transcriptional programs involved in prostate tumorigenesis evidenced by increased AR expression. However, even at 90 weeks of age mice showed only PIN, but no signs of PCa, suggesting that secondary events are required for full cancer progression. Thus, similar to many other prominent genetic alterations in PCa (e.g., affecting *TP53* or *ERG*), KMT2C inactivation is insufficient to initiate malignant transformation.

### Mutant KMT2C Drives Metastatic Transformation in a *Pten-*null Mouse Model of Prostate Cancer

**Fig. 2:**
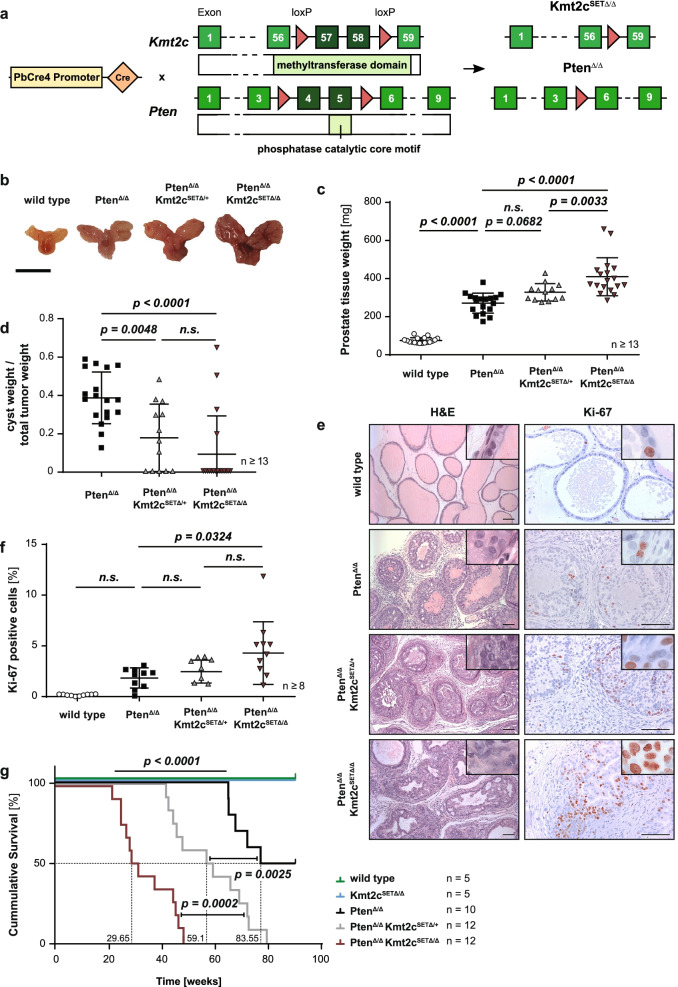
Loss of the *Kmt2c* SET domain exacerbates prostate cancer tumorigenesis in *Pten*-null mice. **a** Scheme of the constructs allowing for the double deletion of *Kmt2c* (top) and *Pten* (bottom) in combination with expression of *Cre* recombinase under the control of the androgen-regulated *probasin* promoter. Mutated transcripts expressed specifically in the prostate epithelium after recombination are depicted on the right-hand side. **b** Representative pictures of prostates of wild type, Pten^∆/∆^, Pten^∆/∆^Kmt2c^SET∆/+^ and Pten^∆/∆^Kmt2c^SET∆/∆^ mice resected at 19 weeks p.p. Scale bar: 10 mm. **c** Quantification of prostate tissue weight of wild type, Pten^∆/∆^, Pten^∆/∆^Kmt2c^SET∆/+^ and Pten^∆/∆^Kmt2c^SET∆/∆^ prostates at 19 weeks p.p. (*n* ≥ 13) **d** Percentage of cystic fluid per total tumour weight (solid tissue and fluid) of Pten^∆/∆^, Pten^∆/∆^Kmt2c^SET∆/+^ and Pten^∆/∆^Kmt2c^SET∆/∆^ prostates at 19 weeks p.p. (*n* ≥ 13) **e** Representative pictures of H&E (left panel) and Ki-67 (right panel) IHC staining of wild type, Pten^∆/∆^, Pten^∆/∆^Kmt2c^SET∆/+^ and Pten^∆/∆^Kmt2c^SET∆/∆^ prostates at 19 weeks p.p., Scale bars: 100 µm. **f** Quantification of cells positive for Ki-67 using QuPath software. (*n* ≥ 8) **g** Kaplan Meier cumulative survival analysis of wild type, Kmt2c^SET∆/∆^, Pten^∆/∆^, Pten^∆/∆^Kmt2c^SET∆/+^ and Pten^∆/∆^Kmt2c^SET∆/∆^ mice (*n* ≥ 5). Values next to the dotted lines at the x-axis of the graph indicate the median life expectancy. *P* values were determined by log-rank (Mantel-Cox) tests. **(c, d, f)** Individual biological replicates are shown. Data are plotted as mean ± standard deviation, and *P* values were determined by ordinary one-way ANOVA with Tukey’s multiple comparisons test

To identify genes with a potential additive effect to *KMT2C* mutations in PCa progression, we analysed the MSKCC/DFCI PCa patient cohort and found a significant co-occurrence of alterations in *PTEN* amongst other genes (Supplementary Fig. 2a). *PTEN* is the most prominent tumour suppressor gene in PCa [20]. Deleterious alterations of this gene are found in ~ 14% of primary and over 30% of metastatic PCa [21]. Previous studies have shown that prostate-specific deletion of *Pten* is sufficient to induce tumorigenesis in mice and that disease progression closely mimics early human PCa [22–24]. Therefore, we back-crossed a mouse model in which conditional deletion of *Pten* is possible [24] to our Kmt2c^SET∆/∆^ animals to generate a double transgenic line with prostate-specific loss of both *Kmt2c-SET* and *Pten* (Pten^∆/∆^Kmt2c^SET∆/∆^) (Fig. 2a).

Prostate-specific deletion of these genes was verified at the genetic level by PCR (Supplementary Fig. 2b). Efficient abrogation of *Pten* mRNA expression levels were found for both Pten^∆/∆^ and Pten^∆/∆^Kmt2c^SET∆/∆^ prostates (Supplementary Fig. 2c). Deletion of the *Kmt2c* SET domain resulted in successful depletion of its full-length transcript. Expression levels of the mutant *Kmt2c* gene in Pten^∆/∆^Kmt2c^SET∆/∆^ double transgenic mice were comparable to full-length *Kmt2c* in Pten^∆/∆^ mice (Supplementary Fig. 2e). We observed no compensatory upregulation of *Kmt2d* upon *Kmt2c* SET domain deletion (Supplementary Fig. 2f). In line with the H3K4 mono-methyltransferase activity of KMT2C we found a global reduction of the enhancer mark H3K4me1 but not H3K27ac in Pten^∆/∆^Kmt2c^SET∆/∆^ prostate tissue (Supplementary Fig. 2g-h). Prostates of mice sacrificed at 19 weeks p.p. were significantly enlarged in Pten^∆/∆^Kmt2c^SET∆/∆^ animals compared to Pten^∆/∆^ controls (Fig. 2b-c). Interestingly, heterozygous deletion of *Kmt2c-SET* in combination with loss of *Pten* (Pten^∆/∆^Kmt2c^SET∆/+^) was sufficient to induce increased prostate weight indicative of tumour development (Fig. 2c).

**Fig. 3:**
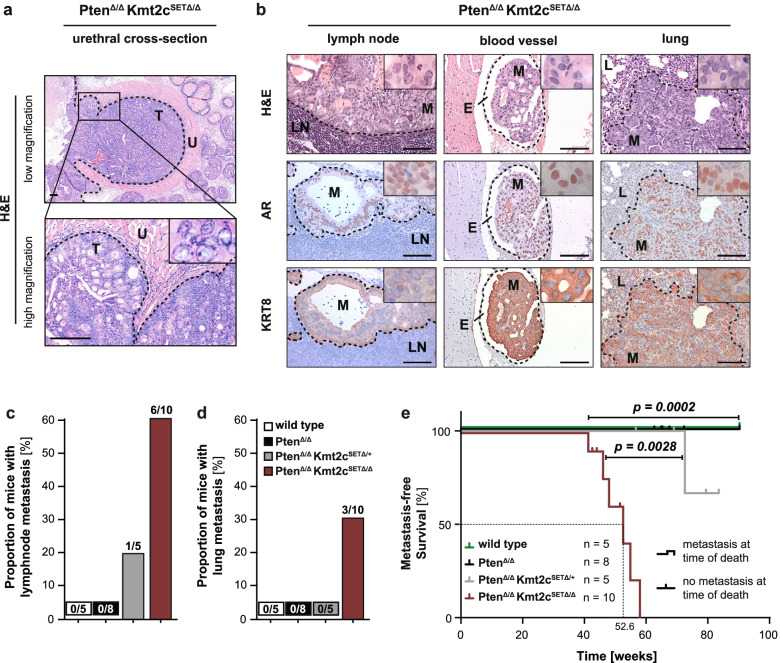
KMT2C methyltransferase deficiency triggers metastatic dissemination of prostate cancer. **a** H&E stains of urinary tract obstruction by invasive PCa in a moribund Pten^∆/∆^Kmt2c^SET∆/∆^ mouse at 39 weeks p.p. at low (top) and high (bottom) magnification. Scale bars: 200 µm. T, tumour; U, urethra. **b** H&E-stained sections and IHC analysis of the androgen receptor (AR) and keratin 8 (KRT8) of tumour dissemination into lumbar lymph nodes, blood vasculature and lungs. Depicted material was derived from two moribund animals at 46- and 48-weeks p.p*.* Scale bars: 100 µm. LN, lymph node; L, lung; E, endothelial lumen; M, metastasis. **c-d** Percentage of wild type, Pten^∆/∆^, Pten^∆/∆^Kmt2c^SET∆/+^ and Pten^∆/∆^Kmt2c^SET∆/∆^ mice presenting with lymph node (**c**) and lung (**d**) metastasis at the time of death. The number above the bar indicates metastasis-bearing/total number of analysed mice (*n* ≥ 5). **e** Kaplan Meier analysis depicting wild type, Pten^∆/∆^, Pten^∆/∆^Kmt2c^SET∆/+^ and Pten^∆/∆^Kmt2c^SET∆/∆^ mice having developed metastasis at the time of death (*n* ≥ 5). Mice that did not present with metastasis at the time of death are shown as censored events. The value below the dotted line indicates the median metastasis-free survival of Pten^∆/∆^Kmt2c^SET∆/∆^ mice. The *P* value was determined by a log-rank (Mantel-Cox) test

We found a striking difference in the gross morphology between Pten^∆/∆^Kmt2c^SET∆/∆^ and Pten^∆/∆^ tumours. Deletion of *Pten* alone in the prostate epithelium led to formation of cystic tumours comprised of ~ 40% fluid, while Pten^∆/∆^Kmt2c^SET∆/∆^ animals developed solid tumours with cyst formation observed only in a minority of mice (Fig. 2d). Histopathological analysis of the primary tumour tissue revealed locally invasive areas in Pten^∆/∆^Kmt2c^SET∆/∆^ animals compared to age matched Pten^∆/∆^ mice. In Pten^Δ/Δ^Kmt2c^SETΔ/Δ^ prostates the basal cell layer was focally disrupted, and isolated tumour cells infiltrated into the surrounding stroma (Fig. 2e, Supplementary Fig. 2i). Double transgenic tumour cells infiltrating into blood vessels or nerve sheaths were observed at localised areas (Supplementary Fig. 2j). Furthermore, we found significantly more Ki-67 positive cells in Pten^∆/∆^Kmt2c^SET∆/∆^ tumours compared to Pten^Δ/Δ^ controls (Fig. 2e-f). Strikingly, Pten^∆/∆^Kmt2c^SET∆/∆^ and even Pten^∆/∆^Kmt2c^SET∆/+^ mice showed a significant reduction in median life expectancy to 30 and 59 weeks, respectively, compared to > 80 weeks for Pten^∆/∆^ animals (*p* < *0.0001*; Fig. 2g). Pten^∆/∆^ and Pten^∆/∆^Kmt2c^SET∆/+^ mice developed large tumours and had to be sacrificed due to tumour burden. In contrast, the general health of Pten^∆/∆^Kmt2c^SET∆/∆^ mice deteriorated rapidly after sudden disease onset despite significantly smaller tumour sizes. Necropsies of Pten^∆/∆^Kmt2c^SET∆/∆^ mice showed invasion of tumour cells into the urethra and urinary bladder, leading to severe obstructive uropathy with parenchymal reduction in the kidneys due to hydronephrosis (Fig. 3a, Supplementary Fig. 3a). This was accompanied by a sharp increase in serum creatinine levels, suggestive of renal failure (Supplementary Fig. 3b). These findings indicate a switch from organ confined PCa towards aggressive disease spreading beyond the prostate upon ablation of KMT2C activity in combination with PTEN loss.

To further investigate the invasive nature of Pten^∆/∆^Kmt2c^SET∆/∆^ prostate tumours we analysed draining lymph nodes and organs to identify metastatic tumour cells in mice showing clinical signs over 40 weeks of age. The prostatic origin of potential metastatic cells was determined by IHC staining of AR and keratin 8 (KRT8) (Fig. 3b). Lymph node metastases were found in more than 50% of analysed Pten^∆/∆^Kmt2c^SET∆/∆^ and 20% of Pten^∆/∆^Kmt2c^SET∆/+^ mice (Fig. 3b-c). Alongside lymphatic metastasis, prostatic tumour cells were detected in the blood vessels demonstrating metastatic dissemination via the circulatory system (Fig. 3b). Remarkably, 3 out of 10 Pten^∆/∆^Kmt2c^SET∆/∆^ mice had further progressed to develop lung metastases (Fig. 3b, d). In contrast, metastatic dissemination could not be found in lymph nodes or in distant organs in Pten^∆/∆^ mice up to 90 weeks p.p*.*, which is in agreement with previously published data [25, 26] (Fig. 3c-d). Kaplan Meier analysis, whereby an event is defined as the presence of metastatic disease detected on necropsy, showed a median time of metastasis-free survival of 52.6 weeks in Pten^∆/∆^Kmt2c^SET∆/∆^ animals (Fig. 3e). This suggests that a significant number of animals likely die due to renal failure from bladder obstruction, resulting from invasive growth of PCa into the local surrounding tissues, before distant metastases of a detectable size can form. Taken together, these data reveal that loss of KMT2C histone methyltransferase activity in *Pten-*null prostate tumours not only drives proliferation, but also confers invasive properties allowing tumour cells to invade surrounding tissues and spread to distant organs.

### Transcriptional Profiling of KMT2C Mutated Prostate Epithelial Cells Reveals Enrichment of the Proliferative MYC Gene Signature

**Fig. 4:**
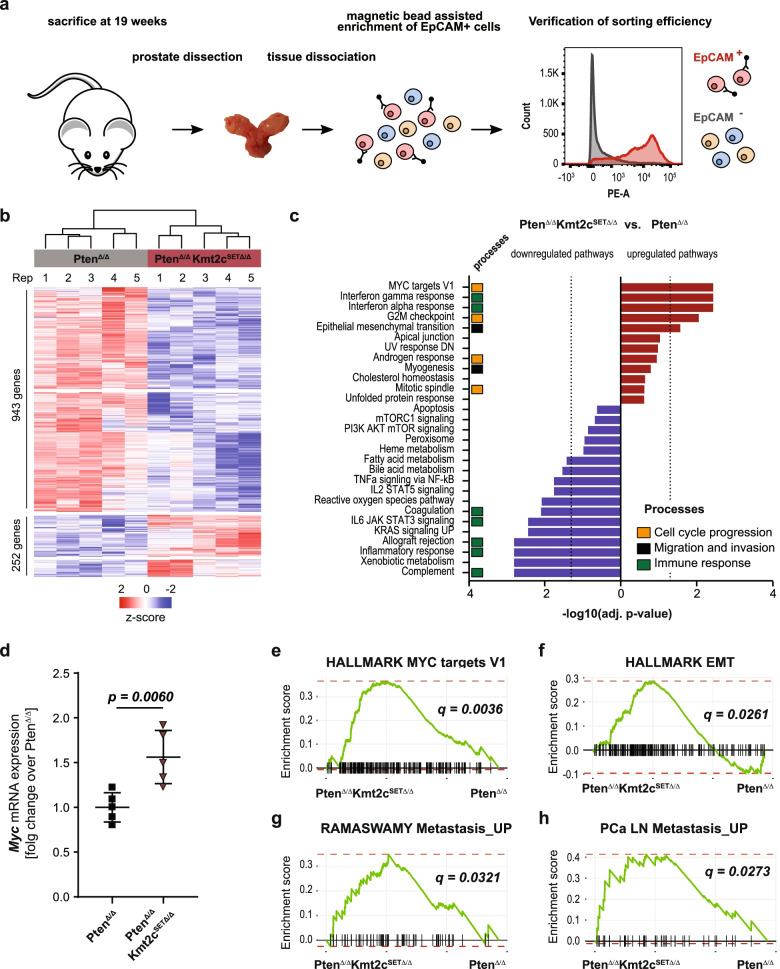
Mutant KMT2C results in enrichment of the MYC gene signature in prostate epithelial cells. **a** Schematic overview of the magnetic bead-based enrichment procedure of murine prostate epithelial cells. Sequence from left to right: 19-week-old mice were sacrificed and prostates were dissected. Tissue was enzymatically and mechanically dissociated to generate single cell suspensions. Cells were labelled with biotinylated anti-EpCAM antibody and retrieved from the bulk population using streptavidin-coated magnetic beads. The sorting efficiency was verified via flow cytometry. **b** Unsupervised hierarchical clustering and heatmap of significant differentially expressed genes between Pten^∆/∆^ and Pten^∆/∆^Kmt2c^SET∆/∆^ prostate epithelial cells. 5 biological replicates were included per group. Number of genes deregulated with an adj. *P* value < 0.05 and log_2_FC ≥ 1 / ≤ -1 are shown on the left. **c** HALLMARK gene sets enriched in Pten^∆/∆^Kmt2c^SET∆/∆^ versus Pten^∆/∆^ groups at an FDR < 0.25. Dotted lines: adj. *P* value = -log10(0.05). **d** Gene expression levels of *Myc* based on normalized counts from RNA-Seq analysis of Pten^∆/∆^ and Pten^∆/∆^Kmt2c^SET∆/∆^ prostate epithelial cells. Individual biological replicates are shown. Data are plotted as mean ± standard deviation, and the *P* value was determined by unpaired two-tailed Student’s t-test. **e–h** fGSEA plots of Pten^∆/∆^Kmt2c^SET∆/∆^ versus Pten^∆/∆^ groups showing an enrichment of MYC target genes (HALLMARK_MYC_TARGETS_V1) (**e**), genes involved in EMT (HALLMARK_EPITHELIAL_MESENCHYMAL_TRANSITION) (**f**), genes upregulated in metastasis of solid tumours (RAMASWAMY_METASTASIS_UP) (**g**) and genes upregulated in prostate cancer lymph node metastasis versus primary prostate cancer (PCa LN Metastasis UP, see also Supplementary Materials and Methods) (**h**)

We performed RNA sequencing (RNA-Seq) of prostate tissue from wild type, Kmt2c^SETΔ/Δ^, Pten^Δ/Δ^ and Pten^Δ/Δ^Kmt2c^SETΔ/Δ^ mice at 19 weeks p.p. following the onset of tumour formation to gain insights into alterations in gene expression that are associated with the observed phenotypes. Cells expressing the epithelial cell adhesion molecule (EpCAM) were isolated from prostate tissue using a magnetic bead-based cell sorting approach. Successful enrichment of EpCAM^pos^ cells was confirmed using fluorescence activated cell sorting (FACS) (Fig. 4a) before RNA isolation and RNA-Seq was conducted. Principal component analysis revealed a clear separation of tumour samples (Pten^Δ/Δ^ and Pten^Δ/Δ^Kmt2c^SETΔ/Δ^) according to their genotypes. In contrast, Kmt2c^SETΔ/Δ^ samples clustered closely with wild type samples, indicating only minor changes in overall gene expression between these groups (Supplementary Fig. 4a). These results substantiate our previous data, highlighting the importance of KMT2C in tumour progression rather than initiation. Whereas differential gene expression analysis of the transcriptomes of Pten^Δ/Δ^Kmt2c^SETΔ/Δ^ with *Pten*-null prostate epithelial cells showed significant up-regulation of 252 and down-regulation of 943 genes (Fig. 4b) the number of significantly deregulated genes between the wild type and Kmt2c^SETΔ/Δ^ cells was considerably smaller with only 5 up- and 80 downregulated transcripts in Kmt2c^SETΔ/Δ^ mice (Supplementary Fig. 4b). To gain further insight into the pathways affected by these gene expression changes, we performed fast pre-ranked gene set enrichment analysis (fGSEA) using hallmark gene sets derived from MSigDB [27, 28]. In accordance with our previous results, we found a significant enrichment for genes driving proliferation upon loss of the KMT2C methyltransferase domain (Fig. 4c, Supplementary Fig. 4c). Of note, we found the proto-oncogene *Myc*, which is a known master regulator of proliferation, and its downstream targets *Ccnd1* and *Ccnd2* to be upregulated in Pten^Δ/Δ^Kmt2c^SETΔ/Δ^ samples (Fig. 4d, Supplementary Fig. 4d). In line with this observation, MYC target genes were overrepresented in *Kmt2c* SET domain deleted samples (Fig. 4e, Supplementary Fig. 4e). Overexpression of MYC in the progression of PCa has previously been observed [8, 29]. Additionally, MYC expression has been shown to correlate with increased disease severity and to positively regulate the well described PCa driver AR [30, 31]. In line with this dependency, we observed an upregulation of androgen response genes in Pten^Δ/Δ^Kmt2c^SETΔ/Δ^ samples (Supplementary Fig. 4f). Besides its role in cellular proliferation, MYC has been recognized as regulating epithelial-to-mesenchymal (EMT) transition and in the general acquisition of invasive properties of cancer cells [32]. Accordingly, an enrichment of genes involved in EMT was observed upon *Kmt2c* SET domain deletion (Fig. 4f, Supplementary Fig. 4 g). To further confirm the invasive nature of Pten^Δ/Δ^Kmt2c^SETΔ/Δ^ prostate epithelial cells we analysed two additional sets of genes described in general to be upregulated in metastatic tumours (RAMASWAMY Metastasis_UP) [33] or lymph node metastasis in PCa (PCa LN Metastasis_UP) [34] and found both signatures to be enriched in the double transgenic samples (Pten^Δ/Δ^Kmt2c^SETΔ/Δ^) compared to controls (Fig. 4g-h). Taken together, changes in the transcriptome observed upon disruption of KMT2C methyltransferase activity verify the activation of proliferative signalling pathways and indicate alterations in processes involved in disease aggressiveness.

### Mutant KMT2C Impairs Oncogene-Induced Expression of the Cell Cycle Repressor p16^INK4A^

**Fig. 5:**
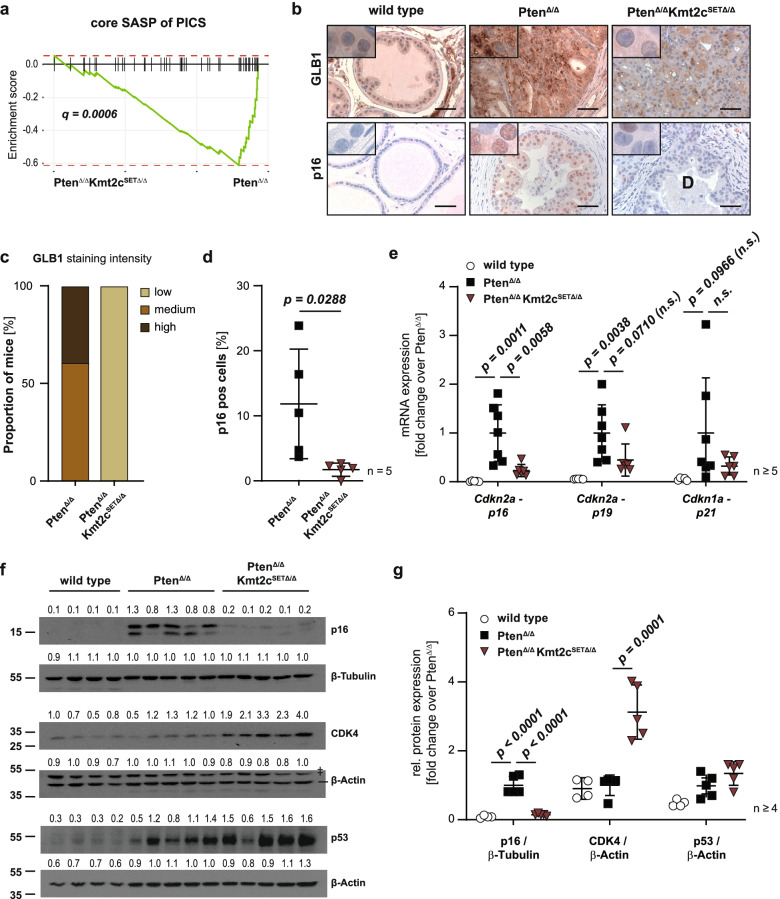
KMT2C SET domain is crucial for oncogene-induced expression of the cell cycle repressor p16^INK4A^. **a** fGSEA plot of Pten^∆/∆^Kmt2c^SET∆/∆^ versus Pten^∆/∆^ transcriptomes showing a depletion of the core SASP gene signature previously described upon PICS in Pten^∆/∆^Kmt2c^SET∆/∆^ samples (core SASP of PICS, see also Supplementary Materials and Methods). **b** IHC staining for GLB1 and p16^INK4A^ on prostate tissue of 19-week-old wild type, Pten^∆/∆^ and Pten^∆/∆^Kmt2c^SET∆/∆^ mice. Scale bars: 50 µm. **c** Semi-quantitative analysis of GLB1 staining intensity performed by a board-certified pathologist. Expression was classified as low, medium, or high of 5 biological replicates analysed per group. **d** Quantification of cells positive for p16^INK4A^ using QuPath software (*n* = 5). **e** RT-qPCR based quantification of *Cdkn2a transcript variant 2* (*p16)*, *Cdkn2a transcript variant 1* (*p19)* and *Cdkn1a* (*p21)* mRNA transcripts (*n* ≥ 5). *P* values of statistically non-significant results are included in the graph for *P* < 0.1 and are additionally labelled as non-significant (n.s.) **f** Western blot analysis showing protein levels of p16^INK4A^, CDK4 and p53 for wild type, Pten^∆/∆^ and Pten^∆/∆^Kmt2c^SET∆/∆^ prostate lysates (*n* ≥ 4). β-Actin and β-Tubulin serve as loading controls. The number above the band depicts the fold change over the average expression level detected in Pten^∆/∆^ samples. ǂ indicates unspecific bands. **g** Quantification of Western blots shown in Fig. 5f. (**d-e, g**) Individual biological replicates are shown. Data are plotted as mean ± standard deviation, and *P* values were determined by unpaired two-tailed Student’s t-test (**d**) or ordinary one-way ANOVA with Tukey’s multiple comparisons test (**e**, **g**)

While sustained growth-stimulatory signalling is a fundamental trait of cancer cells, the replicative stress induced by excessive proliferation may elicit a counteractive growth arrest. This tumour suppressive process, known as oncogene-induced senescence (OIS), can present a barrier to malignant transformation of precursor lesions and must be overcome for tumours to progress to lethal, metastatic disease [35, 36]. Elevated signalling by strong oncogenic drivers, as well as loss of potent tumour suppressors, such as PTEN, have been described to trigger OIS, highlighting the potentially crucial role of this phenomenon in our model system [35, 37]. The unrestrained proliferation and metastatic dissemination of Pten^Δ/Δ^Kmt2c^SETΔ/Δ^ prostate tumour cells described so far indicate an escape from PTEN-loss induced cellular senescence (PICS) in KMT2C mutant tumours. Another key attribute of senescent cells is the extensive change in expression of inflammatory cytokines and other signalling molecules known as the senescence-associated secretory phenotype (SASP) [38]. Interestingly, analysis of the RNA-Seq data (Fig. 4) revealed a significant deregulation of immune response pathways between the transcriptomes of Pten^Δ/Δ^ and Pten^Δ/Δ^Kmt2c^SETΔ/Δ^ prostate epithelial cells (Fig. 4c). This might reflect changes to the SASP as there is considerable overlap of genes involved in those processes. To further investigate potential loss of the SASP gene signature in Pten^Δ/Δ^Kmt2c^SETΔ/Δ^ prostate cells we performed fGSEA using a set of genes previously described to be induced upon PICS (“core SASP of PICS”) [39] and found them to be strongly downregulated in the Pten^Δ/Δ^Kmt2c^SETΔ/Δ^ double transgenic group compared to Pten^Δ/Δ^ mouse prostate cells (Fig. 5a). Next, we performed IHC staining of mouse prostate tissue taken from mice at 19 weeks p.p. for the senescence markers β-galactosidase (GLB1) and p16^INK4A^. Pten^Δ/Δ^ prostate cells showed an accumulation of GLB1 and pronounced induction of p16^INK4A^ which corroborates previously published data describing senescence in the Pten^Δ/Δ^ PCa mouse model [37]. In contrast, expression of both markers was lower in Pten^Δ/Δ^Kmt2c^SETΔ/Δ^ prostate samples (Fig. 5b-d). As senescence is known to be mediated by the two isoforms of *Cdkn2a*, p16^INK4A^ and p19^ARF^, and the *Cdkn1a* gene product p21^CIP1^ [38] we analysed expression of these genes via RT-qPCR. In line with the induction of senescence upon loss of *Pten,* expression of *Cdkn2a* transcripts and, to a lesser extent, *Cdkn1a* was induced in Pten^Δ/Δ^ samples. Combined loss of *Pten* with the *Kmt2c* SET domain led to the loss of p16^INK4A^ expression and a similar but less pronounced effect for p19^ARF^ (Fig. 5e). Western blot analysis for p16^INK4A^ and the cyclin-dependent kinase CDK4, which is known to be inhibited by p16^INK4A^, confirmed the loss of p16^INK4A^ detected in Pten^Δ/Δ^Kmt2c^SETΔ/Δ^ protein lysates and an upregulation of CDK4. In addition, we analysed p53 expression, the key mediator of p19^ARF^-p53-p21^CIP1^-mediated senescence but did not detect a deregulation of this axis at the protein level nor at the transcript level as indicated by no significant change in p21 levels (Fig. 5e-g). We therefore conclude that mutant KMT2C impairs the induction of OIS in *Pten*-null mice by circumventing p16^INK4A^-mediated cell cycle arrest.

### KMT2C Truncation Mutations are Associated with Increased Proliferation and Reduced Disease-Free Survival for Prostate Cancer Patients

**Fig. 6:**
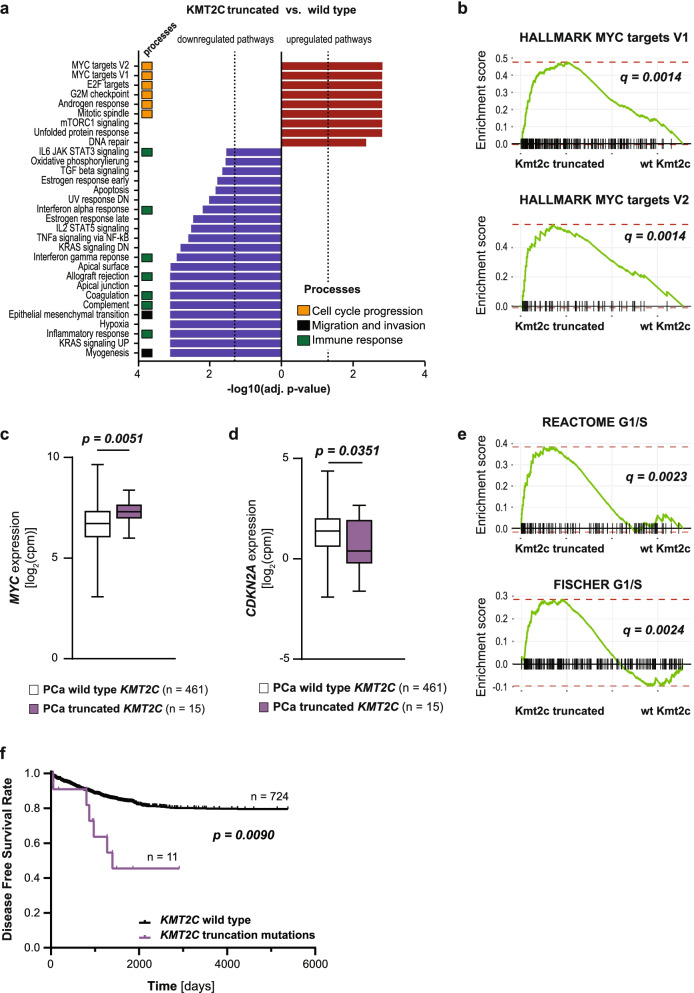
KMT2C truncations enhance MYC target gene expression and correlate with reduced DFS for PCa patients. **a** HALLMARK gene sets enriched in patients with *KMT2C* truncation mutations versus wild type forms of *KMT2C*. Gene sets enriched at an FDR < 0.05 are depicted. Dotted lines: adj. *P* value = -log10(0.05). **b** fGSEA plots of *KMT2C* truncated versus *KMT2C* wild type (wt) prostate cancer patient samples showing an enrichment for MYC target genes (HALLMARK_MYC_TARGETS_V1, HALLMARK_MYC_TARGETS_V2). **c-d** *MYC* (**c**) and *CDKN2A* (**d**) mRNA expression levels in prostate cancer patients with wild type (*n* = 461) or truncated (*n* = 15) forms of *KMT2C*. Data are shown as box and whiskers (min to max) plots, and *P* values were determined by a Mann–Whitney test for non-normal distribution (**c**) or a two-tailed unpaired Student’s t-test (**d**). **e** fGSEA plots of *KMT2C* truncated versus *KMT2C* wild type (wt) prostate cancer patient samples showing an enrichment of transcriptional signatures upregulated during G1/S phase transition (REACTOME_G1_S_TRANSITION, FISCHER_G1_S_CELL_CYCLE). **f** Disease free survival for prostate cancer patients dependent of the *KMT2C* mutational status. Frameshift and nonsense mutations were grouped as truncation mutations (wild type *KMT2C*: *n* = 724; truncated *KMT2C*: *n* = 11). Samples with alterations that were not classified as truncation mutations were excluded from the analysis. Data were retrieved from the ICGC Data Portal and analysed by a log-rank (Mantel-Cox) test. **(a-e)** Data were derived from the TCGA-PRAD dataset

We have identified KMT2C SET domain deletion as a driving event of metastatic PCa in our murine model system and have observed an enrichment of the MYC gene signature accompanied by concurrent loss of p16^INK4A^ as mechanistic downstream effects. Therefore, we hypothesized that *KMT2C* truncation mutations may be associated with aggressive disease and poor prognosis in PCa patients. Therefore, we stratified sample data derived from the Cancer Genome Atlas Prostate Adenocarcinoma (TCGA-PRAD) [20] patient cohort into *KMT2C* wildtype and *KMT2C* truncated tumours for further pathway analysis. Analogous to our murine data, fGSEA of hallmark gene sets revealed strong upregulation of proliferative pathways, with MYC target genes ranking as the most highly enriched gene sets (Fig. 6a-b). Furthermore, mRNA expression of *MYC* itself was upregulated in patient samples carrying *KMT2C* truncation mutations (Fig. 6c). In line with the capacity of MYC to induce transcription and enhance the stability of the AR, we detected enrichment of androgen response genes in the *KMT2C* truncated patient group (Supplementary Fig. 5a). As our murine data show that activation of proliferative signalling pathways occurs concurrently with evasion of p16^INK4A^-mediated growth arrest, we performed fGSEA on the “core SASP of PICS” gene set and found a significant reduction of this transcriptional signature in *KMT2C* truncated tumours (Supplementary Fig. 5b), potentially indicating loss of senescence features. We further observed a reduction in expression of the gene encoding p16^INK4A^, *CDKN2A*, in these samples (Fig. 6d). As p16^INK4A^ inhibits cell cycle progression at the G1/S transition, we performed fGSEA of two sets of genes upregulated during this process (REACTOME G1/S, FISCHER G1/S) and found these to be highly enriched in the *KMT2C* truncated patient group, further highlighting the likely depletion of cell cycle repressors in this cohort (Fig. 6e). Collectively, these data indicate hyperactivation of proliferation in PCa carrying truncated forms of KMT2C, which is remarkably similar to the effect observed in our model system. Thus, we hypothesize that *KMT2C* mutations, in agreement with our murine data, also influence prognosis in PCa. Therefore, we analysed data derived from the International Cancer Genome Consortium (ICGC) [40] and found that *KMT2C* truncation mutations (nonsense and frameshift mutations) correlate with reduced disease-free survival (DFS) in PCa (Fig. 6f). Taken together our data show that KMT2C truncating events drive cancer progression by upregulating MYC target genes and circumventing p16^INK4A^-mediated growth arrest. These effects may ultimately facilitate the formation of lethal, metastatic disease in PCa patients. Therefore, *KMT2C* truncation mutations represent a biomarker for aggressive disease and indicate that inhibitors of the MYC signalling axis might be beneficial to these patients.

## Discussion

Although KMT2C is the most frequently mutated epigenetic regulator in PCa [15], and loss of histone methylation correlates with a poorer clinical outcome [41], functional studies investigating the impact of these mutations on prostate carcinogenesis are scarce. Here, we describe for the first time that loss of the KMT2C methyltransferase domain accelerates tumour growth and promotes a switch from indolent to lethal, metastatic disease in vivo when combined with other PCa associated mutational events, specifically loss of PTEN. Our study also reveals that this exacerbation of tumour progression is accompanied by an enrichment of the proliferative MYC gene signature and an escape of p16^INK4A^-mediated oncogene-induced senescence.

Previous studies have shown correlations between expression levels of KMT2C and cancer progression, while others have instead highlighted the importance of genetic alterations affecting protein function [17, 42–44]. To date, the contribution of KMT2C to carcinogenesis has most extensively been studied in breast cancer (BCa), which is biologically similar to PCa due to its dependence on steroid hormone signalling. Reduced expression of KMT2C or KMT2D results in decreased oncogenic estrogen receptor (ER) signalling, possibly due to impaired interaction with the ER pioneer factor Forkhead box protein A1 (FOXA1) [44, 45]. In contrast, low KMT2C expression correlates with bad prognosis in BCa [43] and both truncation mutations as well as mutations in the plant homeodomain (PHD) of KMT2C have been shown to be tumour-promoting events [17, 44]. Thus, the contribution of KMT2C to carcinogenesis appears to be influenced by cellular context, alteration type, as well as hormone-dependence even within a single malignancy. Our analysis of the mutational spectrum in PCa patients showed a high prevalence of nonsense and frameshift mutations resulting in truncated forms of KMT2C, especially in metastatic samples. We therefore focused our work on the genetic loss of the C-terminal methyltransferase domain rather than the analysis of expressional changes.

To study the complex biological consequences of KMT2C alterations in vivo*,* we established a genetically engineered mouse model (GEMM) with a prostate epithelium specific deletion of the catalytic core motif of *Kmt2c*. *Kmt2c*-mutated animals developed PIN lesions but never progressed to develop carcinomas. This observation resembles other GEMMs modelling key alterations observed in human PCa, such as deletion of *TP53* or overexpression of ERG or AR in the prostate epithelium [25, 46, 47]*.* Regardless, loss of the *Kmt2c* SET domain resulted in the activation of the oncogenic AR signalling pathway and a significant increase in cellular proliferation, albeit at low levels. These data are in contrast to the ability of wildtype KMT2C/D in facilitating the closely related ER signalling axis in BCa [44, 45]. A potential difference in interaction of ER and AR with their co-regulators, including FOXA1, might result in diverging effects of KMT2C on these transcriptional programs. However, the varied effects of KMT2C observed across different BCa model systems hints at a multitude of factors capable of influencing the biological outcome of KMT2C alterations in carcinogenesis.

Previous studies have identified loss of PTEN or overexpression of MYC to be sufficient to induce carcinogenesis in vivo. However, neither alteration leads to metastatic disease suggesting a requirement for other contributory genetic events [23, 48]. Formation of distant metastasis has previously been achieved in the *Pten*-null model in combination with select additional genetic alterations [26, 39, 49–51]. For example, it was recently shown that the combined overexpression of MYC and loss of *Pten* in prostate luminal epithelial cells can induce lethal metastatic PCa [52]. Our analysis of the mutational spectrum present in PCa patients revealed a significant co-occurrence of *PTEN* and *KMT2C* alterations, pointing towards a synergistic effect. In accordance, we demonstrate in our study that *Kmt2c* SET domain deletion represents an as-yet unknown aberration capable of initiating the important switch from localized to metastatic PCa in a *Pten*-null GEMM.

A frequent observation in relation to metastatic transformation is the circumvention of the senescent phenotype initiated upon *Pten*-deficiency [37], or upregulation of the proto-oncogene MYC [50–52]. There is ample evidence for the fundamental role of senescence as an abortive stage of cancer development. However, its impact on tumorigenesis appears to be diverse and highly dependent on the context in which it arises. A multitude of inducers of senescence have been described, yet the molecular effectors activated downstream mainly converge on two major tumour suppressive pathways: the p16^INK4A^-RB and the p14^ARF^-p53-p21^CIP1^ signalling axes [53]. Interestingly, genetic deletion of *p19*^*Arf*^, the murine homolog to *p14*^*ARF*^, in *Pten*-null mice does not impact p53 expression in vivo and is not sufficient to abrogate senescence or promote tumorigenesis [54]. Furthermore, while senescent features are lost in *Pten / p53* double-knockout prostate epithelial cells in a GEMM, the formation of distant metastases was not observed [25]. These findings indicate that circumvention of OIS may be insufficient to drive metastatic transformation and needs to occur in parallel with the induction of an additional driver of cellular dissemination. In our model system, combined deletion of *Pten* and the *Kmt2c* catalytic core motif led to loss of p16^INK4A^ in double transgenic mice but failed to convincingly show any change in the p19^ARF^-p53-p21^CIP1^ axis. Still, we observed a loss of senescence features and, more importantly, a drastic reduction in life expectancy due to obstructive tumour cell infiltration into the urethra and the development of lymph node and lung metastases. Our data additionally suggest a possible involvement of the MYC signalling axis in the switch to metastatic PCa in our model system based on increased expression of MYC target genes and an upregulation of *Myc* mRNA levels. However, as other drivers of proliferation might activate a similar set of downstream targets and MYC protein levels and activity are tightly controlled not only on transcriptional level but also through RNA stability, protein turnover and posttranslational modifications [55] the transcriptional changes observed in our model system may not exclusively point towards MYC but drivers of proliferation in general.

Based on the functional role of KMT2C as an H3K4 mono-methyltransferase, loss of p16^INK4A^ might directly be regulated by impaired placement of activating H3K4me1 marks at enhancer elements associated with *Cdkn2a*. On the other hand, an upregulation of *Myc* is unlikely to be a direct effect of H3K4me1 loss and possibly mediated by the downregulation of a transcriptional inhibitor of *Myc* instead. While our data convincingly show a global deregulation of H3K4me1 upon impaired KMT2C methyltransferase activity deciphering site specific changes in the enhancer landscape in our model system and how they correlate with transcriptional changes remains to be investigated.

The relevance of our findings to human patients is supported by the fact that we observe both enrichment of the MYC gene signature and downregulation of *CDKN2A,* which encodes p16^INK4A^, in PCa patients with truncation mutations of *KMT2C*. Remarkably, we found a significant correlation between DFS and truncated *KMT2C* in patients, demonstrating the prognostic significance of *KMT2C* mutation status in PCa. Based on our findings, we speculate that inhibition of MYC transcriptional activity may be a viable treatment option for patients with *KMT2C* truncation mutations. Furthermore, elucidating the complex transcriptional networks that are altered in response to *KMT2C* mutations uncovered in this study may facilitate the identification of novel pro-metastatic pathways and promote the development of new clinical therapeutics that can counteract PCa metastases.

Taken together, this work reveals new insights into the previously poorly understood transition from local to lethal metastatic PCa. Furthermore, data presented in this study provide a rationale for the inclusion of *KMT2C* mutation status in standard diagnostics of patients with suspected PCa. Based on our findings, inhibition of MYC-associated transcriptional activity could represent a strategy for treating PCa patients with deleterious *KMT2C* mutations and thus a poor prognosis. As many other human cancers also show a high *KMT2C* mutation prevalence our work may have translational relevance to an array of malignancies similarly affected.

## Methods

### Analysis of mutation data from publicly available datasets

Mutation data including frequency and co-occurrence of specific mutations in PCa patients were obtained from the MSKCC/DFCI dataset using http://www.cbioportal.org [15, 56, 57].

The DFS Rate of PCa patients dependent on *KMT2C* mutational status was derived from the ICGC Data Portal. For “*KMT2C* wildtype” all patients with *KMT2C* mutations of any kind, including those of unknown significance, were removed. Mutations of the consequence type “Frameshift” and “Stop Gained” were grouped as truncation mutations. Consequence type “Missense” was selected for missense mutations. All available PCa projects (PRAD-US, PRAD-CA, PRAD-UK, EOPC-DE, PRAD-CN, PRAD-FR) were included in this analysis.

### Animal models

Pb-Cre4 (Tg(Pbsn-cre)4Prb, RRID: IMSR_JAX:026,662), Pten^flox/flox^ (Pten^tm2Mak^, RRID: IMSR_RBRC02300) and Kmt2c^flox/flox^ (Kmt2c^tm1.2Jwle^, RRID: MGI:4,948,101) mice have previously been described [18, 19, 24]. All animals were maintained on a C57Bl/6-Sv/129 mixed background. Male mice were allocated to experimental groups based on their genotype and sacrificed at 19 or 90 weeks of age as indicated in the individual figure legends. All animal studies were reviewed and approved by the Federal Ministry Republic of Austria for Education, Science and Research and conducted according to regulatory standards (BMBWF GZ 66.009/0135-WF/V/3b/2016).

### Pathologic review

Sections were reviewed by two independent board-certified pathologists with specific expertise in laboratory animals (L.K. and S.H.). All analyses were performed blinded to genotype.

### Immunohistochemistry and histological analysis

Immunohistochemistry (IHC) and haematoxylin/eosin stains (H&E) were performed with formalin-fixed paraffin-embedded (FFPE) tissue using standard protocols. The following antibodies were used for IHC: Ki67 (Cell Signaling, #12,202, 1:400), AR (Abcam, ab133273, 1:100), p16 (Abcam, ab211542, 1:500), GLB1 (Novus Biologicals, NBP2-45,731, 1:120), KRT8 (Abcam, ab53280, 1:100) and p63 (Abcam, ab735, 1:80).

All images were taken with a Zeiss AxioImager M2. Quantification was performed using QuPath 0.1.2 [58]. If automated quantification was not possible, semi-quantitative analysis was performed by a board-certified pathologist with specific expertise in mouse models of prostate cancer (L.K.). Analyses were performed blinded to genotype.

### Western blot analysis

Protein extraction from frozen prostate samples for Western blot analysis has previously been described [49]. Western blots were prepared using 15–20 µg of protein lysate, blocked with 5% BSA in 1 × TBS / 0.1% Tween-20 for 1 h and incubated at 4 °C overnight with primary antibodies against AR (1:1000, ab133273, Abcam), p16^INK4A^ (1:2000, ab211542, Abcam), CDK4 (1:200, sc-260, Santa Cruz), p53 (1:1000, CST#2524, Cell Signaling), β-Actin (1:1000, CST#4967, Cell Signaling) and β-Tubulin (1:2000, CST#2146, Cell Signaling). Western blots were quantified using ImageJ2.

### RNA Isolation and RT-qPCR

RNA from mouse tissue was extracted using TRI Reagent (Merck) and purified using the ReliaPrep RNA Tissue Miniprep kit (Promega). DNase digestion was performed on a column. For RT-qPCR, 1 µg of total RNA was reverse transcribed to cDNA using the iScript cDNA Synthesis Kit (Bio-Rad). RT-qPCR was performed in triplicates with the Luna Universal qPCR Master Mix (NEB). All procedures were performed according to the manufacturers’ instructions. Real-time monitoring of PCR amplification was performed using the CFX96 Real-Time System C1000 Thermal Cycler (Bio-Rad). mRNA levels were calculated using the Pfaffl analysis method [59] and normalized to the geometric mean of β-actin and cyclophilin A. RT-qPCR primer sequences are listed in the Supplementary Materials and Methods.

### RNA-Seq of mouse prostate epithelial cells

Mice were sacrificed at 19 weeks p.p. and prostates were dissected. Prostate tissue was digested as previously described [60]. Cell suspensions were strained through a 40 µm cell strainer and washed twice using MACS buffer (1 × PBS + 2 mM EDTA + 2% FCS). The cell solution was subject to centrifugation for 5 min at 150 g, and cells were resuspended in 800 µL MACS buffer. Cells were passed 5 times through a 27G needle and counted. 1–3 * 10^7^ cells were collected by centrifugation at 300 g for 5 min and resuspended in 200 µL MACS buffer. 100 ng anti-CD326-biotin (13–5791-82, eBioscience) were added to the cell solution and incubated at room temperature for 10 min. 1 mL MACS buffer was added to the solution and cells were collected by centrifugation at 300 g for 5 min. The cell pellet was resuspended in 200 µL MACS buffer and transferred to a 12 × 75 mm FACS tube. 60 µL streptavidin positive selection beads (T9424, Sigma) were added to the tubes and incubated for 10 min at room temperature. 2.5 mL MACS buffer were added to the cell-bead suspension and the tube was transferred to a magnetic stand. Beads were allowed to adhere to the magnet for 5 min at room temperature before MACS buffer was discarded. Beads were washed twice more in an identical manner using 2.5 mL MACS buffer for each wash and resuspending the cell-bead solution with a P1000 pipette between washes. The bound fraction containing CD326-positive cells was then collected in 5 mL MACS buffer and subject to centrifugation for 5 min at 150 g at 4 °C. Cells were resuspended in 200 µL MACS buffer, counted, and transferred to a fresh (RNase-free) Eppendorf tube. 5 µL of sorted prostate epithelial cells were removed for FACS analysis. The remaining cells were pelleted at 300 g for 5 min at 4 °C, supernatant was removed, and the cell pellet was snap frozen in liquid nitrogen and stored at -80 °C until further use.

RNA isolation was performed using the ReliaPrep RNA Tissue Miniprep kit (Promega). RNA quality was assessed using the 4200 TapeStation System (Agilent). For library preparation, the Ultra II Directional RNA Library Prep Kit (E7760, NEB) was used in combination with a poly(A) mRNA magnetic isolation module (E7490) and multiplex oligos for Illumina (E7600). Library preparation was performed according to the manufacturer’s instructions (optimized for an insert size of 200 nt, input: 250 ng, adaptor dilution: fivefold, 11 amplification cycles). The library quality was analysed using the 4200 TapeStation System.

### RNA-Seq data analysis

Raw FASTQ files from murine RNA-Seq were subject to a quality check with FastQC (v0.11.5) [61] and MultiQC (v1.4) [62]. Adapters and low-quality read ends were trimmed using Trimmomatic (v0.36) [63] and reads shorter than 35 nt were discarded. The pre-processed reads were mapped to the reference mouse genome (Ensembl GRCm38) utilizing gene annotation (Ensembl v91) with STAR [64, 65]. The quality of mapping was evaluated with RSeQC (v2.6.4) [66] and Picard (v2.10.6) [67] and rRNA content was checked with FastQ Screen (v0.13.0) [68]. Gene quantification was performed on uniquely mapped reads only, with featureCounts (v1.5.2) [69]. Differential expression analysis was carried out in R (version 3.5.1) [70] with DESeq2 package (v1.20.0) [71] and limma package (v3.38.2) [72]. Genes with a FDR-adjusted *P* value < 0.05 and fold change ≥ 2 or fold change ≤ 0.5 were considered significantly differentially expressed. Heatmaps of differentially expressed genes (DEG) were generated using unsupervised hierarchical clustering with the pheatmap package (v1.0.12) [73]. fGSEA of gene sets was done with the fgsea package (v1.14.0) [74]. Gene sets were derived from MSigDB (version 6.1.1) (hallmark gene sets, Ramaswamy_metastasis_up, Reactome_G1_S_transition, Fischer G1_S_cell_cycle) or previously published works (core SAPS of PICS [37], PCa LN metastasis [34]). Gene sets based on previously published works are detailed in the Supplementary Materials and Methods. Human gene symbols present in custom gene sets were converted to orthologous mouse genes using the biomaRt package (v2.44.4) [75].

### TCGA data analysis

Harmonized TCGA PRAD [20] RNA-seq data were acquired as HTSeq‐Counts via R package TCGAbiolinks (v2.16.4) [76]. Differentially expressed genes between patients with truncated and wild-type forms of *KMT2C* were identified with DESeq2 package (v1.20.0) [71]. fGSEA of hallmark gene sets (MSigDB version 6.1.1) and custom gene sets was performed with the fgsea package (v1.14.0) [74]. Detailed steps and parameters of the analysis are described in the Supplementary Materials and Methods.

### Statistical analysis

Data in figures are plotted as individual replicates with their mean and standard deviation for analyses with n ≤ 20 per group or as boxplots depicting the 25^th^ to 75^th^ percentile (box) and min to max (whiskers) for *n* > 20 per group. DFS rate for data derived from the ICGC database was calculated using the “cohort comparison” tool provided by the platform. All other statistical analyses were performed using GraphPad Prism 8. The significance level of differences between groups was determined by two-tailed unpaired Student’s t-tests for 2 groups or ordinary one-way ANOVA with Tukey’s multiple comparisons tests for 3 or more groups. For Kaplan–Meier analysis the log rank (Mantel-Cox) test was performed.

## Supplementary Information

**Additional file 1:** **Supplementary Figure 1.** related to Figure 1. **Supplementary Figure 2.** related to Figure 2. **Supplementary Figure 3.** related to Figure 3. **Supplementary Figure 4.** related to Figure 4. **Supplementary Figure 5.** related to Figure 6.

**Additional file 2.** Supplementary Methods.

**Additional file 3. Supplementary Table 1.** Genotyping Primers. **Supplementary Table 2.** Genotyping Protocols. Supplementary Table 3. RT-qPCR Primers. **Supplementary Table 4.** Geneset “PCa LN Metastasis UP”. **Supplementary Table 5.** Geneset “core SASP of PICS”.

## Data Availability

The RNA-Seq dataset supporting the conclusions of this article is available in the GEO repository, GSE186413 and is publicly available as of date of publication. The following publicly available datasets were used: The Cancer Genome Atlas—Prostate Adenocarcinoma (https://portal.gdc.cancer.gov/projects/TCGA-PRAD). The long tail of oncogenic drivers in prostate cancer [15], ICGC Prostate Cancer Project (PRAD-US, PRAD-CA, PRAD-UK, EOPC-DE, PRAD-CN, PRAD-FR) [40].

## Abbreviations

AR: Androgen receptor
BCa: Breast cancer
COMPASS: Complex of proteins associated with Set1
DEG: Differentially expressed genes
DFS: Disease-free survival
EMT: Epithelial-to-mesenchymal transition
EpCAM: Epithelial cell adhesion molecule
FACS: Fluorescence activated cell sorting
fGSEA: Fast pre-ranked gene set enrichment analysis
FFPE: Formalin-fixed paraffin-embedded
GEMM: Genetically engineered mouse model
GLB1: β-Galactosidase
H&E: Haematoxylin and eosin
H3K4me1: Mono-methylation of lysine 4 on histone 3
IHC: Immunohistochemistry
ICGC: International Cancer Genome Consortium
KRT8: Keratin 8
PHD: Plant homeodomain
PIN: Prostatic intraepithelial neoplasia
PICS: PTEN-loss induced cellular senescence
p.p.: Postpartum
PbCre4: Probasin Cre promoter
PCa: Prostate Cancer
PCR: Polymerase Chain Reaction
PI3K: Phosphoinositide 3-kinase
qRT-PCR: Quantitative reverse transcription-PCR
PTEN: Phosphate and Tensin Homologue
RNA-Seq: RNA sequencing
SASP: Senescence-associated secretory phenotype
TCGA-PRAD: Cancer Genome Atlas Prostate Adenocarcinoma
