# Supplementary material for: KMT2C methyltransferase domain regulated INK4A expression suppresses prostate cancer metastasis
Source: Mol Cancer. 2022 Mar 30;21:89. doi: 10.1186/s12943-022-01542-8 (PMC8966196; doi:10.1186/s12943-022-01542-8)
Supplement: Supplementary file 1 — Additional file 1: Supplementary Figure 1. related to Figure 1. Supplementary Figure 2. related to Figure 2. Supplementary Figure 3. related to Figure 3. Supplementary Figure 4. related to Figure 4. Supplementary Figure 5. related to Figure 6. [file 12943_2022_1542_MOESM1_ESM.docx]

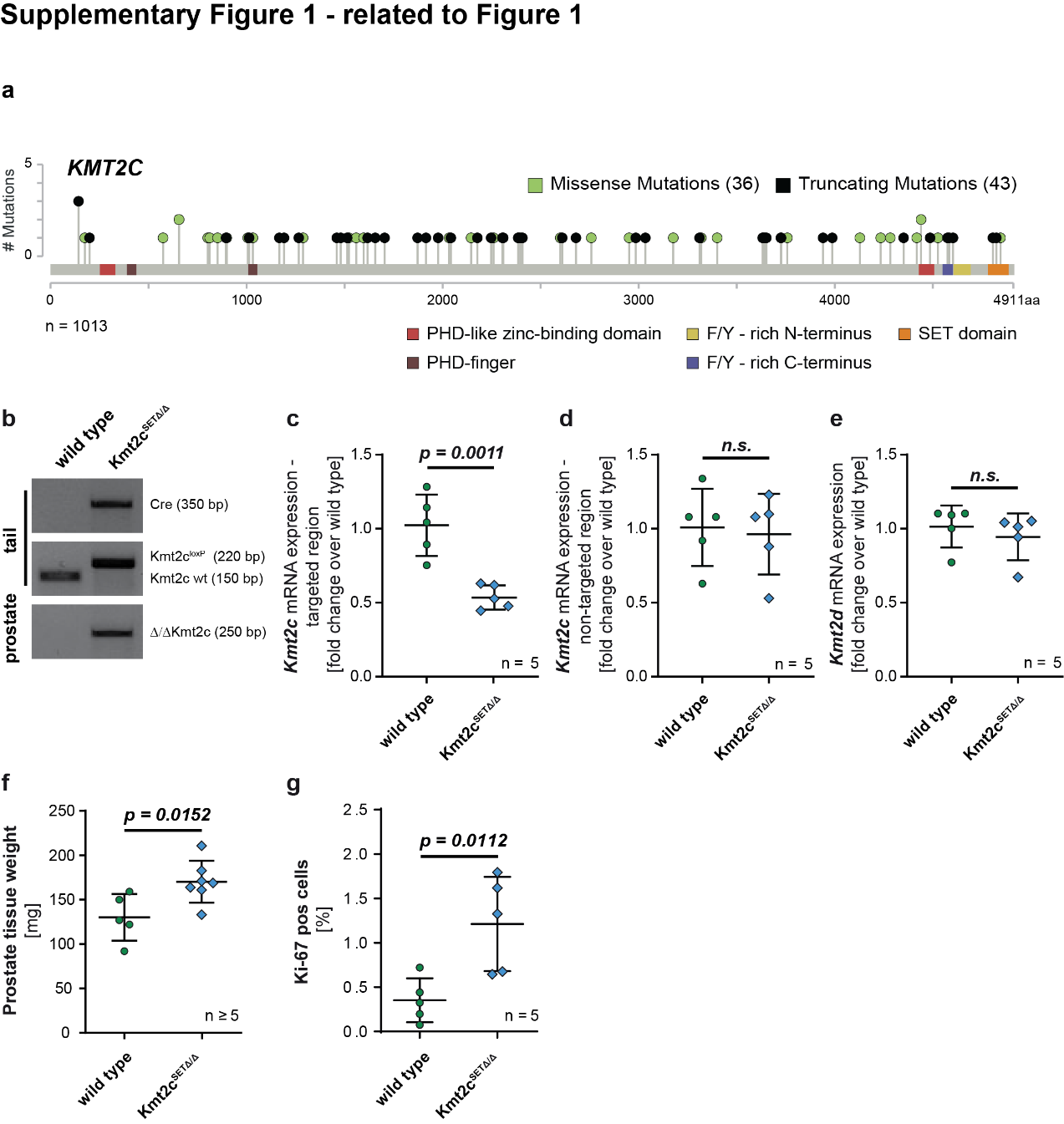


**Suppl. Figure 1.** related to Figure 1. **a,** Diagram of the *KMT2C* gene with indications for mutations identified in the MSKCC/DFCI cohort (n = 1013). **b,** PCR-based analysis of the *Cre* transgene and the *Kmt2c* gene locus in a wild type and a Kmt2c^SET∆/∆^ sample. Depicted are results for *Cre* and the non-recombined *Kmt2c* alleles (without loxP sites: wt; with loxP sites: loxP/loxP) from tail DNA as well as the recombined (∆/∆) *Kmt2c* locus from purified genomic DNA of prostates after *Cre* expression. **c, d, e,** RT-qPCR based quantification of mRNA transcripts normalized to geometric mean of *Actb* and *Ppia* and depicted as fold change over wild type of a targeted (C) and non-targeted (D) *Kmt2c* gene region and mRNA transcripts of *Kmt2d* (E). **f,** Prostate weight of wild type and Kmt2c^SET∆/∆^ mice at 90 weeks p.p. **g,** Quantification of cells positive for Ki-67 in prostates of 90-week-old mice using QuPath software. **c-g,** Individual biological replicates are shown. Data are mean ± SD, and *P* values were determined by unpaired two-tailed Student’s t-tests.


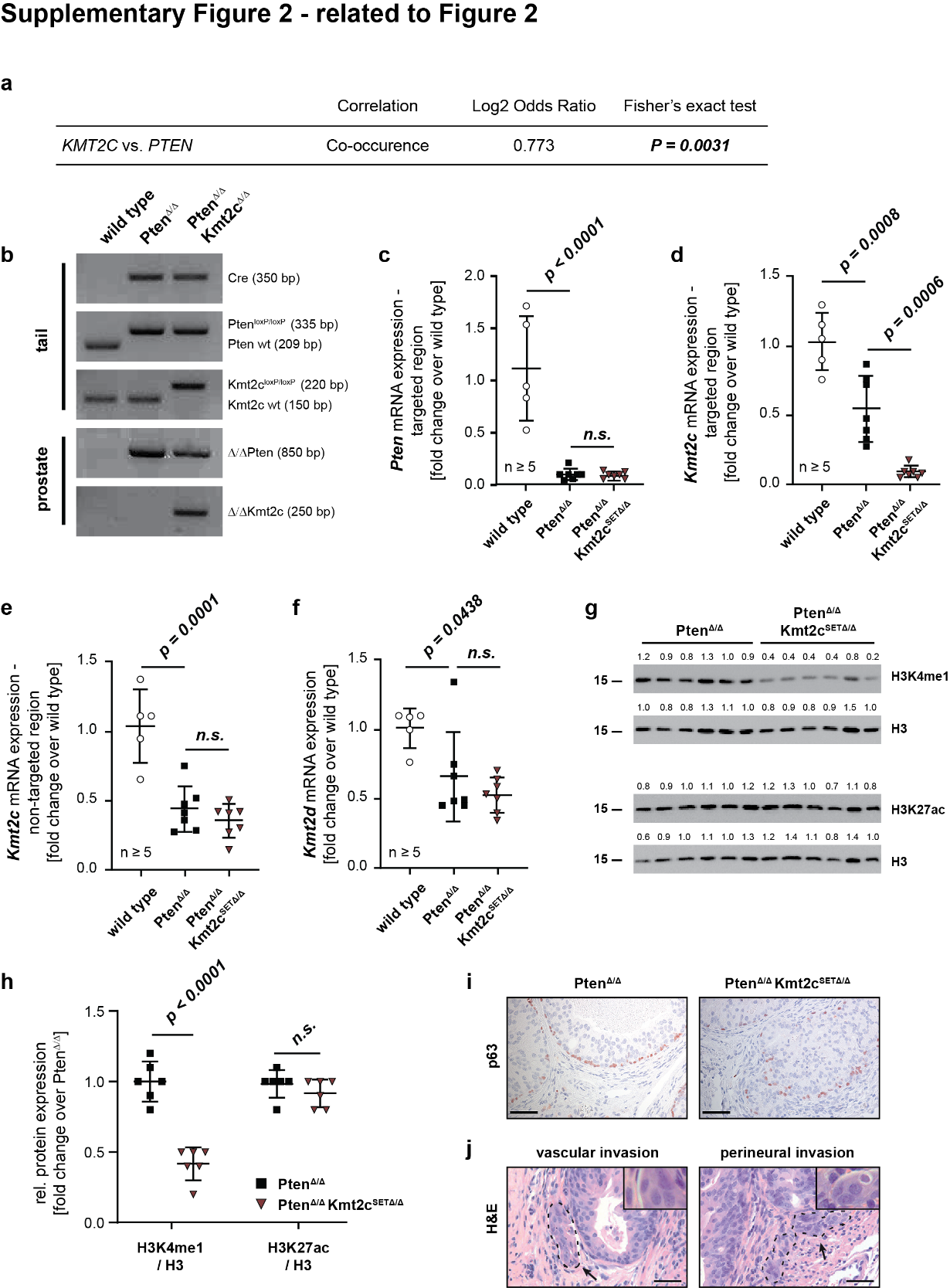


**Suppl. Figure 2.** related to Figure 2. **a,** Fisher’s exact-test analysing co-occurrence of *KMT2C* and *PTEN* mutations in the MSKCC/DFCI PCa patient cohort (n = 1013). **b,** PCR-based analysis of the *Cre* transgene and the *Kmt2c* and *Pten* gene locus in wild type, Pten^∆/∆^ and Pten^∆/∆^Kmt2c^SET∆/∆^ mice. Depicted are results for *Cre* and the non-recombined *Kmt2c* and *Pten* alleles (without loxP sites: wt; with loxP sites: loxP/loxP) from tail DNA as well as the recombined (∆/∆) *Kmt2c* and *Pten* loci from purified genomic DNA of prostates after *Cre* expression. **c-f,** RT-qPCR based quantification of mRNA transcripts normalized to geometric mean of *Actb* and *Ppia* and depicted as fold change over wild type of *Pten* (c), a targeted (d) and non-targeted (e) *Kmt2c* gene region and *Kmt2d* (f). Individual biological replicates are shown. Data are plotted as mean ± standard deviation, and *P* values were determined by ordinary one-way ANOVA with Tukey’s multiple comparisons test. **g**, Histone Blot analysis showing levels of H3K4me1 and H3K27ac for Pten^∆/∆^ and Pten^∆/∆^Kmt2c^SET∆/∆^ prostate lysates (n = 6). H3 served as loading control. The number above the band depicts the fold change over the average expression level detected in Pten^∆/∆^ samples. **h,** Quantification of Histone Blots shown in Fig. S2g. Individual biological replicates are shown. Data are plotted as mean ± standard deviation, and *P* values were determined by unpaired two-tailed Student’s t-test **i,** Representative pictures of p63 IHC stainings of Pten^∆/∆^ and Pten^∆/∆^Kmt2c^SET∆/∆^ mouse prostates at 19 weeks p.p. depicting focal disruption of basal cell layer in Pten^∆/∆^Kmt2c^SET∆/∆^ samples. Scale bars: 50 µm. **j,** Examples of vascular (left) and perineural (right) invasion of tumour cells in Pten^∆/∆^Kmt2c^SET∆/∆^ mouse prostates at 19 weeks p.p. Scale bars: 50 µm.


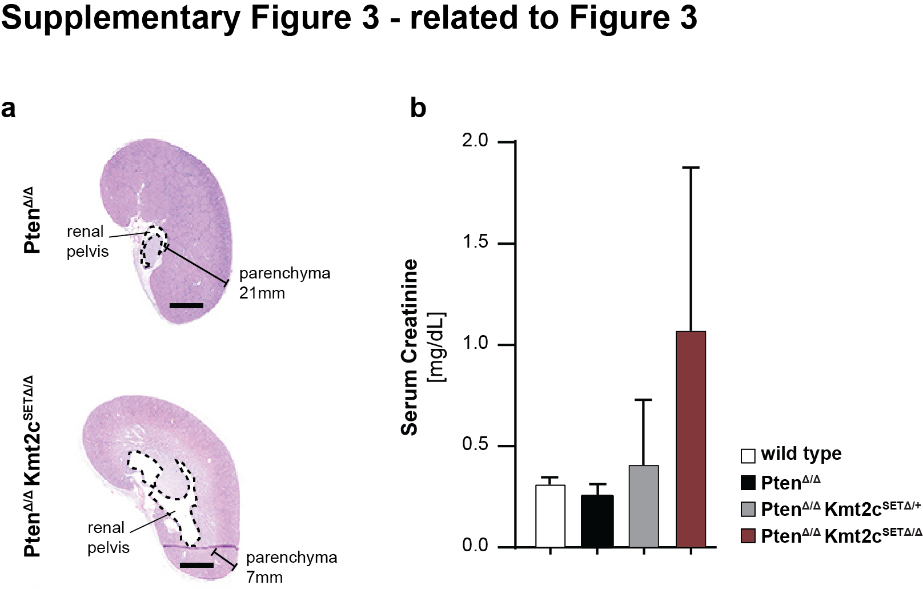


**Suppl. Figure 3.** related to Figure 3. **a,** Whole slide H&E images of massive parenchymal loss and renal pelvic expansion due to hydronephrosis following urinary tract obstruction occurring in a late stage Pten^∆/∆^Kmt2c^SET∆/∆^ mouse and a healthy kidney of an age matched Pten^∆/∆^ control animal. Parenchyma width estimations are shown. Scale bars: 200µm. **b,** Serum creatinine levels of wild type, Pten^∆/∆^, Pten^∆/∆^Kmt2c^SET∆/+^ and Pten^∆/∆^Kmt2c^SET∆/∆^ mice. Serum of wild type animals was collected at 38 weeks p.p*.* for comparison. Serum of tumour-bearing animals was collected at moribundity. Pten^∆/∆^, mean age: 84 weeks p.p.; Pten^∆/∆^Kmt2c^SET∆/+^, mean age 48 weeks p.p., Pten^∆/∆^Kmt2c^SET∆/∆^, mean age 37 weeks p.p.


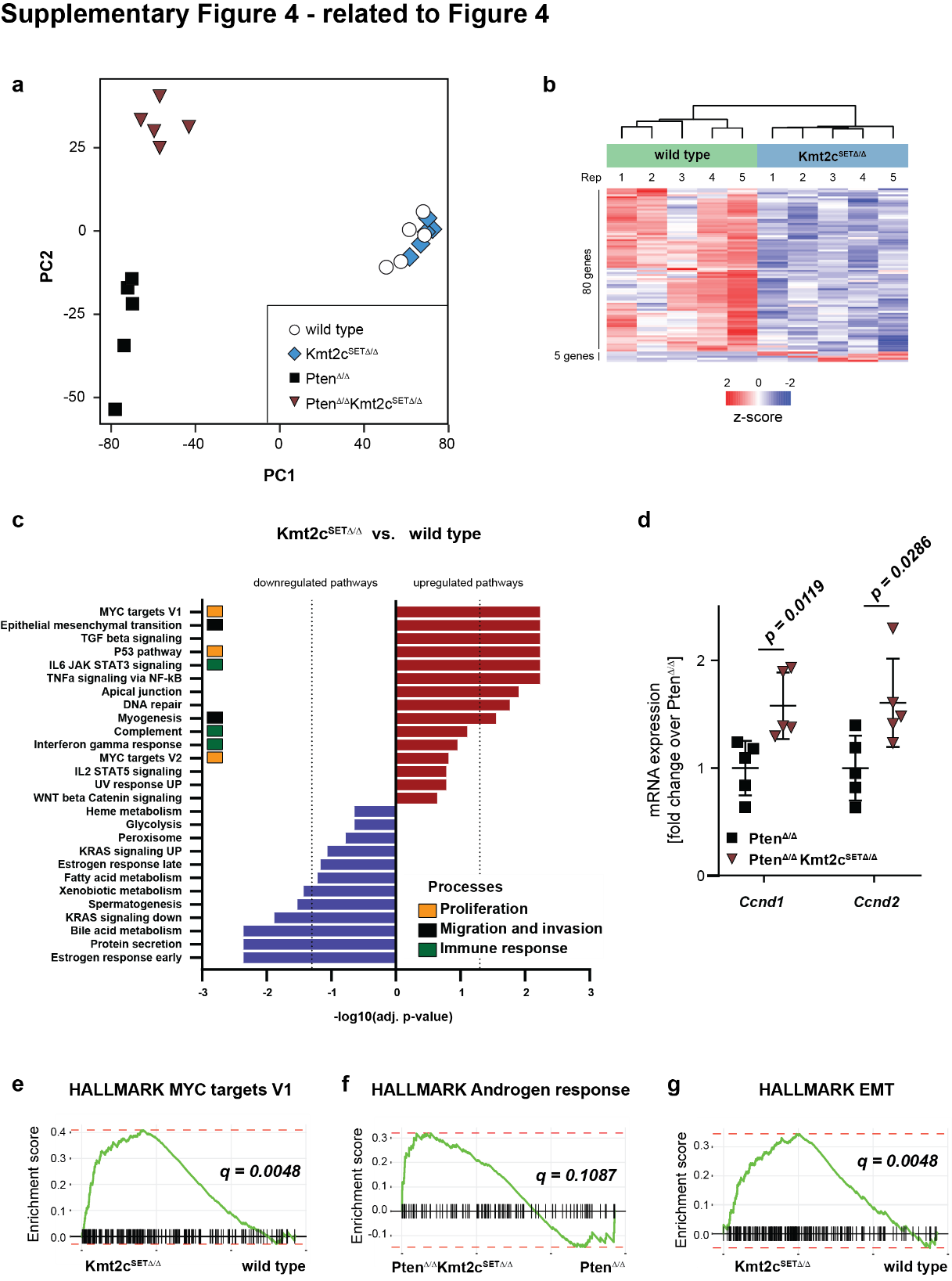


**Suppl. Figure 4.** related to Figure 4. **a,** Principal component analysis (PCA) of mRNA samples of prostate epithelial cells derived from 19-week-old mice. 5 individual biological replicates are shown. **b,** Unsupervised hierarchical clustering and heatmap of significantly differentially expressed genes between wild type and Kmt2c^SET∆/∆^ prostate epithelial cells. Number of genes deregulated at q-value < 0.05 and log_2_FC ≥ 1 / ≤ -1 are shown on the left. **c,** HALLMARK gene sets enriched in Kmt2c^SET∆/∆^ versus wild type groups at an FDR < 0.25. Dotted lines: adj. *P* value = -log10(0.05). **d,** Gene expression levels of *Ccnd1* and *Ccnd2* based on normalized counts from RNA-Seq analysis of Pten^∆/∆^ and Pten^∆/∆^Kmt2c^SET∆/∆^ prostate epithelial cells. Individual biological replicates are shown. Data are plotted as mean ± standard deviation, and *P* values were determined by unpaired two-tailed Student’s t-tests. **e, f, g,** fGSEA plots of Kmt2c^SET∆/∆^ versus wild type groups (e, g) and Pten^∆/∆^Kmt2c^SET∆/∆^ versus Pten^∆/∆^ groups (F) showing an enrichment of MYC target genes (HALLMARK_MYC_TARGETS_V1) (e), androgen response genes (HALLMARK_ANDROGEN_RESPONSE) (f) and genes involved in EMT (HALLMARK_EPITHELIAL_MESENCHYMAL_TRANSITION) (g).


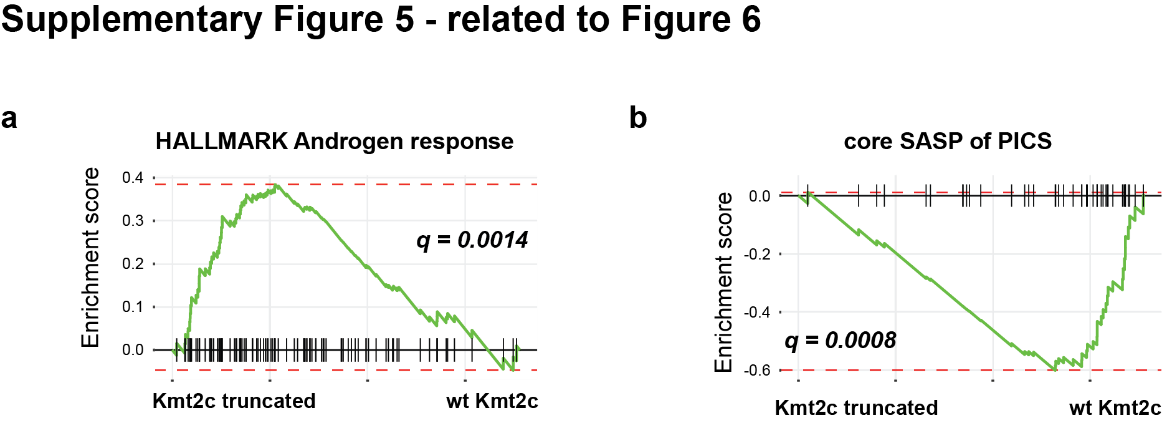


**Suppl. Figure 5.** related to Figure 6. **a, b,** fGSEA plots of *KMT2C* truncated versus *KMT2C* wild type (wt) prostate cancer patient samples showing an enrichment of androgen response genes (HALLMARK_ANDROGEN_RESPONSE) (a) and a depletion of the “core SASP of PICS” gene signature (Core SASP of PICS, see also Supplementary Materials and Methods) (b) in *KMT2C* truncated samples.
