# Supplementary material for: KMT2C methyltransferase domain regulated INK4A expression suppresses prostate cancer metastasis
Source: Mol Cancer. 2022 Mar 30;21:89. doi: 10.1186/s12943-022-01542-8 (PMC8966196; doi:10.1186/s12943-022-01542-8)
Supplement: Supplementary file 2 — Additional file 2. Supplementary Methods. [file 12943_2022_1542_MOESM2_ESM.docx]

**Supplementary Methods**

**DNA isolation and PCR for genotyping**

Ear punch or tail biopsies were digested for 2h in 400µL tail lysis buffer (100mM Tris pH 8.0, 5mM EDTA, 200mM NaCl, 0.2% SDS) and 15µL Proteinase K (10mg/mL) (Fisher Scientific) at 55°C. 170µL 5M NaCl were added, and samples were centrifuged for 5 minutes at 15000 rpm. Supernatant was transferred to new Eppendorf tube and mixed with 500µL isopropanol. DNA was pelleted by centrifugation for 10 minutes at 15000 rpm, supernatant was discarded, and pellets were washed once with 500µL 70% ethanol. Samples were centrifuged for 5 minutes at 15000 rpm, ethanol was removed, and DNA was air-dried for 10 minutes. DNA was reconstituted in 150µL ddH_2_O for 1h at 37°C and stored at 4°C. For determination of genotype 2µL DNA were used per PCR reaction. PCR was performed using OneTaq DNA polymerase (NEB) and dNTP Mix (LabConsulting) in a C1000 Touch Thermal Cycler (Bio-Rad) according to manufacturer’s instructions. Primer sequences and protocols used for genotyping are listed in Supplementary Materials and Methods.

**Histone Isolation**

Prostate samples (~70 mg) were homogenized in 300 µL ice-cold PBS + 10 mM Sodium Butyrate. Cells were spinned down for 5 minutes at 1200 rpm at 4°C and washed once with 1 mL of ice-cold PBS + 10 mM Sodium Butyrate. Pellet was resuspended in 1mL lysis buffer (10 mM Tris pH 6.5, 50mM Sodium Bisulphite, 1% Triton X 100, 10 mM MgCl2, 8.6% Sucrose, 10 mM Sodium Butyrate, Protease Inhibitor (Roche)), vortexed and spinned down for 5 minutes at 2500 rpm at 4°C. Pellet was washed three times with 1 mL lysis buffer and once with histone wash buffer (10 mM Tris pH 7.4, 13 mM Na3EDTA, 10 mM Sodium Butyrate, Protease Inhibitor). 100 µL H2O and 1 µL of concentrated H2SO4 were added to a final concentration of 0.4N. Samples were incubated on ice for 2 hours and centrifuged at 15000 rpm for 10 minutes at 4°C. Histones were precipitated from supernatant with 1 mL ice-cold acetone at -20°C overnight and collected by 15 minutes of centrifugation at 15000 rpm at 4°C. Pellet was washed once in 1 mL acetone, air-dried and resuspended in 20 µL H2O. Histone blots were prepared using 2-3 µg of histone sample, blocked with 5% BSA in 1 x TBS / 0.1% Tween-20 for 1 hour and incubated at 4°C overnight with primary antibodies against H3K4me1 (1:1000, ab8895, Abcam) or H3 (1:1000, CST#4499, Cell Signaling). Histone blots were quantified using ImageJ2.

**Serum Creatinine Assay**

Animals were anesthetized using 100µL anesthesia (Ketamine (5mg/mL) and Xylazine (1.6mg/mL)) per 10g body weight and blood was collected by retro-orbital bleeding. Blood was allowed to clot at room temperature for at least 30 minutes (up to 60 minutes). Samples were then centrifuged at 4°C for 10 minutes at 1500g. Serum was transferred to a new Eppendorf tube and stored at -20°C until analysis. For creatinine quantification Creatinine Assay Kit (MAK080, Sigma Aldrich) was used according to manufacturer’s instructions.
